# Opinion paper: gynecological surgery in local anesthesia?

**DOI:** 10.1007/s00404-022-06572-7

**Published:** 2022-04-29

**Authors:** Felix Neis, Diethelm Wallwiener, Melanie Henes, Bernhard Krämer, Sara Brucker

**Affiliations:** Department of Obstetrics and Gynecology, University Hospital Tübingen, Calwerstrasse 7, 72076 Tübingen, Germany

**Keywords:** Paracervical block, Local anesthesia, Gynecologic surgery without anesthesia

## Abstract

**Purpuse:**

The paracervical block (PCB) is a local anesthesia procedure that can be used to perform gynecological surgeries without the need for further anesthesia. With the PCB, surgeries can be moved from the central operating room to outpatient operating rooms, where they can be performed without the presence of an anesthesia team.

**Methods:**

In this paper, the indications, implementation and limitations of the procedure are discussed.

**Conclusion:**

Especially in times of scarce staff and OR resources during the Corona pandemic, OR capacity can be expanded in this way.

## Introduction

The Corona pandemic has shown that the healthcare system is on the verge of collapse in times of pandemic infections. The shortage worldwide is particularly evident in operating rooms, as anesthesiologists and nurses are being diverted to intensive care units. This may delay necessary operations for the treatment or diagnosis of precancerous or malignant diseases of the female genitals. Hence, a rethinking of surgical gynecology becomes necessary. Office hysteroscopy has already shown that smaller gynecological procedures can be moved out of the operating room if anesthesia capacity is not required for them.

The Viennese ophthalmologist Koller first described the feasibility of performing an eye surgery under local anesthesia with cocaine in 1884 [1]. In the same year, Halsted described the first peripheral nerve block with cocaine. However, the initial euphoria was dampened as early as 1891 by a report of 200 systemic intoxications with 13 deaths [2]. Since then, local anesthetics have continued to evolve.

While most operations in Germany are currently still performed under general anesthesia, a look at other countries shows that the majority of outpatient operations can be performed under local anesthesia [3–6]. Scarce OR resources (operating room, anesthesia team, etc.) in times of the corona pandemic make it reasonable to perform surgeries without the need for an anesthesia team (anesthesiologist and nurse).

In this context, the paracervical block (PCB) enables performing operations in which a pain stimulus is applied in the area of the cervix uteri without the need for general anesthesia. According to the “Consensus Statement for Recommended Terminology Describing Hysteroscopic Procedures” of the International Working Group of the American Association of Gynecologic Laparoscopists (AAGL), the European Society for Gynaecological Endoscopy (ESGE) and the Global Community of Hysteroscopy (GCH) this stands for a level 4 of pain management [7]. Known complications of general anesthesia such as postoperative nausea and vomiting (PONV) or postoperative cognitive dysfunction (POCD) in elderly patients can be avoided.

The skills acquired during office hysteroscopy serve as an introduction to operations with PCB. Surgeries that can be performed include:

- Diagnostic hysteroscopy (usually no analgesia necessary) with and without abrasion.
- Operative hysteroscopy (polyp, myoma, septum, synechiae, etc.).
- Loop electrosurgical excision procedure (LEEP) or laser vaporization of the transformation zone of the uterine cervix.
- Removal of pregnancy tissue.

By using local anesthetics that diffuse quickly to the nerve (lidocaine, mepivacaine), the above procedures can be performed after a short time of exposure to the drug.

### How to make a PCB

Before performing an operation in PCB, there is a detailed communication with the patient. The creation of a pleasant environment, empathic information before the operation and good communication between the surgeon and the patient during the operation are important bases for the successful realization of an operation in PCB [3, 8]. Even before the operation, trust-building measures can pave the way for a successful operation in PCB.

The step-by-step explanation of the operation directly at the operating table has proven to be helpful. In this way, the patient's fears can be eliminated even before the operation. Knowing the sequence of the individual steps reduces the patient's anxiety and makes the operation easier for both the surgeon and the patient. Relaxing music can also be played to ease the preoperative tension [9].

After disinfection of the vagina and vulva, the bladder is emptied using disposable catheters. Alternatively, if the surgeon wishes to avoid catheterization of the bladder, the patient can be asked to go to the toilet directly before entering the operating room. In general, the more space available in the surgical area, the more comfortable the operation is for the patient, as unpleasant pressure, e.g., by using specula, can be avoided for adjustment. After insertion of a speculum, the cervix uteri is hooked with tenaculum at the 12 o'clock position. To stabilize the cervix as well as to distract the patient from the pain stimulus, the patient may be asked to cough. Injection of local anesthetic before hooking the cervix with the tenaculum is usually not necessary. The short pain during hooking is usually better tolerated by the patient than the prick and pressure caused by the injection of local anesthetic.

**Fig. 1:**
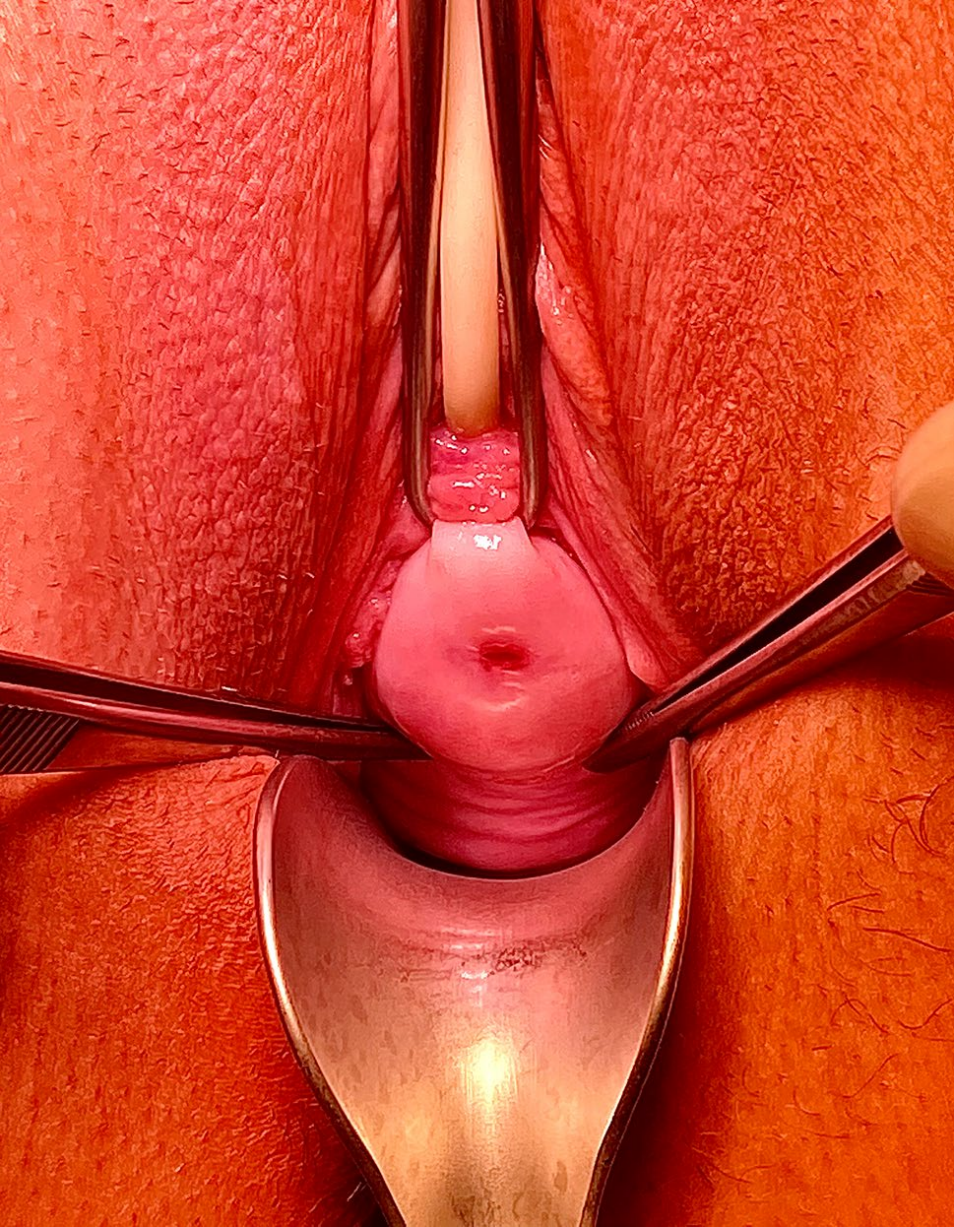
Visualization of the sacrouterine ligaments by pulling the cervix caudally. Two tweezers (5 and 7 o'clock position) show the region of the ligg. sacrouterinae

By pulling the cervix uteri caudally, the sacrouterine ligaments can be visualized properly on both sides (Fig. 1).

The manufacturer's recommendations for performing a PCB are a maximum of 15 ml for lidocaine 1% and a maximum of 6–10 ml per side for mepivacaine 1%. However, one should not rely on the specifications for the maximum dosage of the local anesthetics, since side effects may occur even earlier [10, 11].

**Fig. 2:**
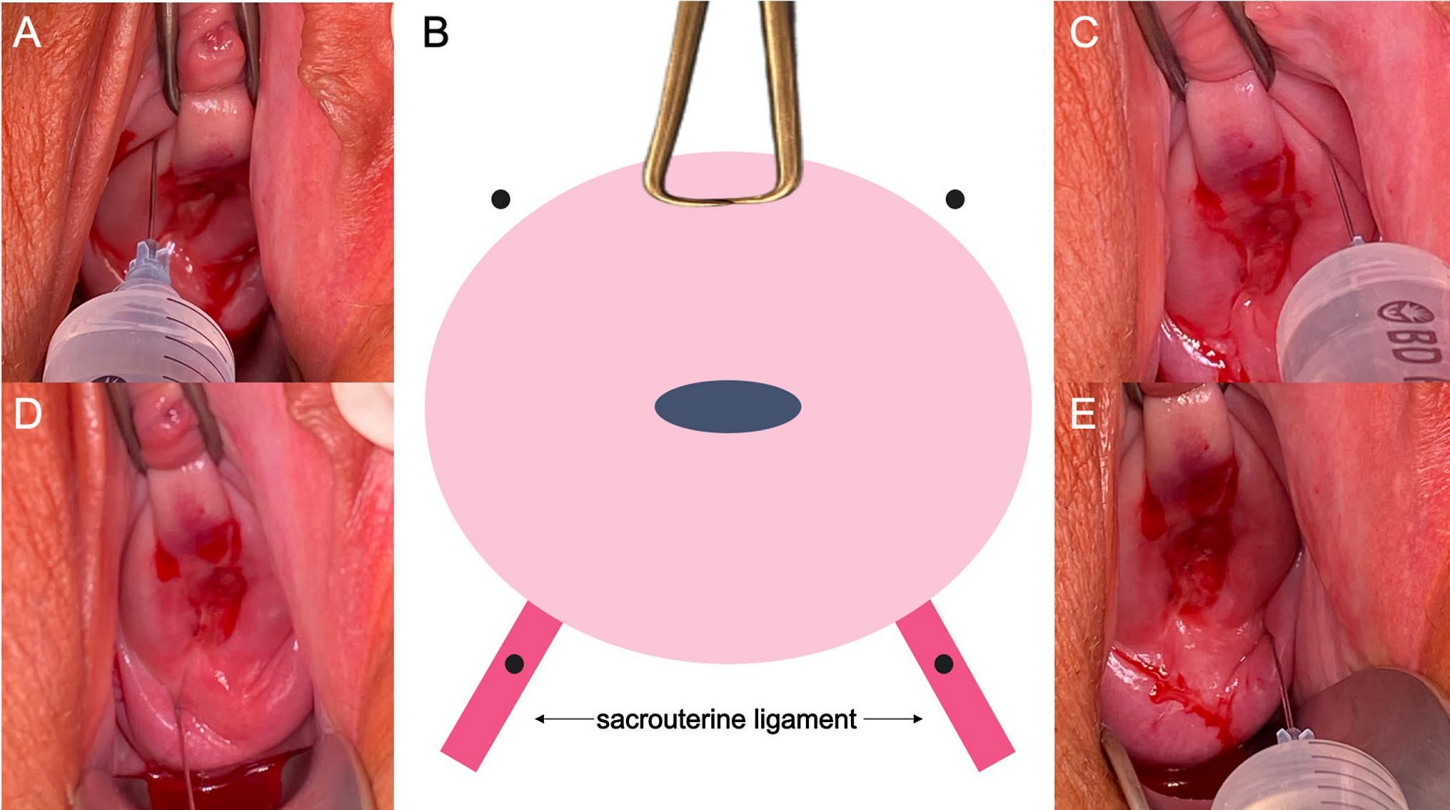
Positioning of the injections for PCB. **A** 11 o'clock position. **B** Schematic drawing of the uterine cervix. Teneculum at 12 o'clock position. Black dots: Injection positions. **C** 1 o'clock position. **D**) Injection position sacrouterine ligament right side. **E** Injection position sacrouterine ligament left side

From our own experience, an injection of 5 ml lidocaine 1% into the region of the sacrouterine ligament on both sides in combination with 3 ml lidocaine at the 1 and 11 o'clock position next the cervix is sufficient for most procedures (Fig. 2). The current literature is inconsistent as to whether the two-point injection technique or the four-point technique with 10 ml lidocaine 1% into both sacrouterine ligaments is preferable [12, 13]. In Tuebingen, the four-point technique is preferred due to good experience. To achieve rapid diffusion to the nerves supplying the cervix, the needle should be inserted to a depth of approximately 2–3 cm [14]. To avoid intravascular injection, aspiration must be performed prior to infiltration. To avoid the side effects of PCB, the injection should be carried out slowly, fractionated and under regular negative aspiration. In case of accidental puncture of a vessel, the injection should be performed only after new placement and negative aspiration. If resistance to injection is too high, the needle should be repositioned. In this case, it is usually sufficient to withdraw the needle by a few millimeters.

**Fig. 3:**
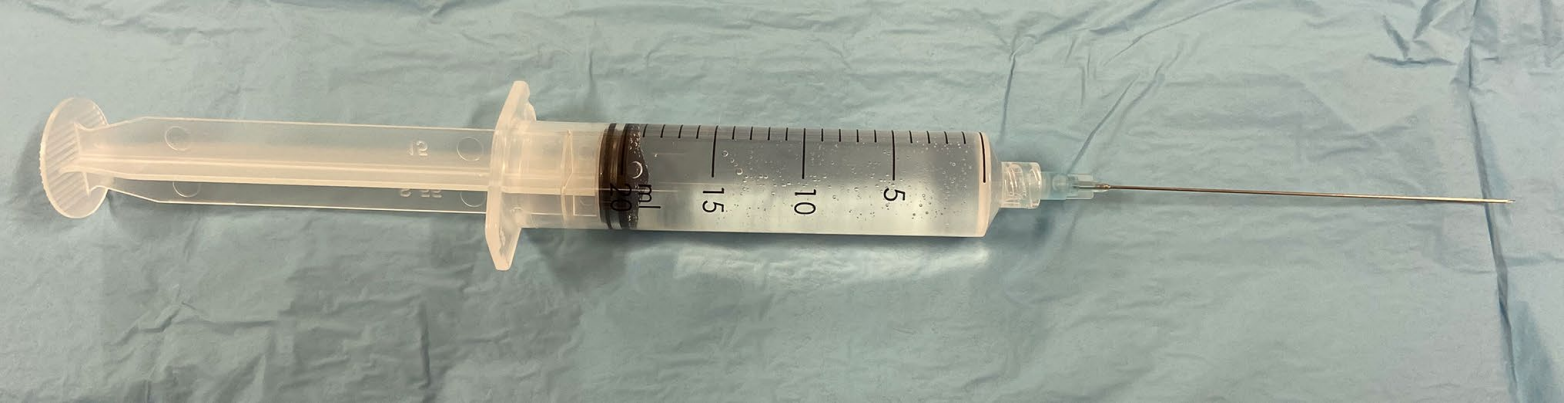
20 ml syringe with Luer Lock connector attached to a 8 cm long 23G injection cannula

Since a total of approximately 16 ml of lidocaine 1% is injected, in the technique proposed above, it is recommended to use a 20 ml syringe with a Luer Lock connection to fix the 23 G injection cannula (Fig. 3). The smaller the injection cannula, the less painful the injection is for the patient. The injection cannula should have a length of at least 80 mm so that the sacrouterine ligaments can be comfortably reached from the vagina without discomfort for the patient.

The time from injection to onset of complete nerve block depends on the characteristics of the used local anesthetic. In addition, the concentration plays an important role: the higher the concentration, the faster is the onset of action [10]. After the injection, a waiting time of 3–4 min must be maintained until the onset of the effect. During the waiting time, a swab soaked with lidocaine 1% can be inserted into the vagina to reduce the sensation of heat during LEEP.

In case of persistent discomfort, especially during dilatation of the cervix or pain during LEEP, a further injection of 1–2 ml lidocaine 1% intracervically may be necessary. Since the nerve fibers are already pre-saturated, only small amounts are necessary during subsequent injections [15]. In this case an additional injection directly into the cervix uteri might be useful. A needle with a larger diameter can be used to compensate the higher resistance of the cervix. For further pain control, especially in psychologically very tense patients, inhalation of nitrous oxide (N_2_O) has been proven to be safe, effective and economical [16]. The administration of i.v. sedation or analgesics is usually not necessary. The administration of nonsteroidal anti-inflammatory drugs (NSAIDs) 60 min before surgery also had a positive effect on intraoperative pain perception. In postoperative pain therapy, orally or rectally applied NSAIDs showed an equally good effect [17]. Intraoperative administration of scopolamine does not appear to have any further analgesic effect [18]. The use of opioids also results in good pain control, but with a significantly higher rate of side effects [17].

### Own results

After office hysteroscopy was an established procedure in our institution, surgeries were increasingly performed in PCB. Initially, procedures were expanded to LEEP conizations for small dysplasias and hysteroscopy with fractionated abrasion for postmenopausal bleeding and endometrial hyperplasia. As experience grew, the procedure was expanded more broadly. Thus, the PCB procedure was expanded with larger polyps and vulvar interventions. Also, the procedure was shared by the experienced surgeons so that currently a pool of 16 surgeons are skilled in the PCB. With further expansion of procedures without anesthesia, over time more and more procedures could be transferred from the central OR to the outpatient OR. While there were only 23 procedures in the first month, we were able to increase the numbers to 51 procedures already in the 2nd month. The number of surgeries in the PCB stabilized around 40–50 surgeries per month, while additional surgeries in local anesthesia (breast surgery, vulva surgery, surgery in the upper layers of the abdominal and thoracic wall) were performed as well.

## Discussion

### Complications

A feared but rare complication of PCB is local anesthetic systemic toxicity (LAST) [10, 11, 19]. It occurs as a potentially life-threatening complication in approximately 0.03% of peripheral regional anesthetics with lipophilic local anesthetics [20]. Local anesthetic-induced toxic systemic side effects are due to blockade of voltage-dependent sodium channels in the CNS and heart. Precursors of LAST are perioral numbness, tinnitus, metallic taste, dizziness or hypotension, change in pulse rate, cardiac arrhythmias [20]. If these signs occur, the injection should be stopped immediately.

Risk factors for LAST include local anesthetic used, extreme age (< 16 years and > 60 years), low muscle mass, female sex, pregnancy, previous cardiac disease, hepatic or renal disease, metabolic disorders, and central venous disease [10, 11, 19, 20]. However, older patients in particular benefit from performing surgery in local anesthesia, as the risk of POCD in patients over 60 years of age is 6.6% [21].

Even if the side effects of local anesthesia are rather rare, precautions for short-term securing of the airway and to perform cardiopulmonary resuscitation should be in place. Emergency medication to therapy of an allergic shock and for resuscitation, as well as midazolam, diazepam for breaking the LAST must be available. Due to the lipophilic characteristic of most drugs used for local anesthesia, early i.v. administration of a lipid infusion has been established when neurological and cardiac side effects occur [19, 20]. This binds the lipophilic local anesthetic and thus weakens its effect [22].

Especially in patients at risk, baseline monitoring (pulsoximetric oxygen saturation, 3-channel ECG, non-invasive blood pressure measurement, i.v. access) should be used to rapidly detect any cardiovascular side effects [15, 19, 20].

Patients should be monitored for 30 min postoperatively to detect late-onset adverse events. Causes of late-onset side effects include high plasma levels due to overdose, rapid absorption, reduced metabolism, or reduced plasma protein binding [23]. If cardiac adverse events occur, the monitoring time should be extended to at least 2 h; if central venous adverse events occur, the monitoring time should be extended to at least 4–6 h [19].

### Patient selection

To perform surgery in PCB, without the presence of an anesthesia team and without the need for an operating room, patient selection is crucial. Relative contraindications are: anxious patient, tight vaginal conditions, large dysplasia areas, cervical stenosis, and intrauterine pathology larger than 1.5–2 cm.

With good preparation and empathy, even the most anxious patient can undergo surgery in PCB. In case of strong fear or already strong pain during the examination before the operation, the general anesthesia is preferable. Communication during surgery is the most important aspect in the use of PCB. Patients in whom there is a language barrier therefore have a higher risk of postoperative dissatisfaction with the procedure.

**Fig. 4:**
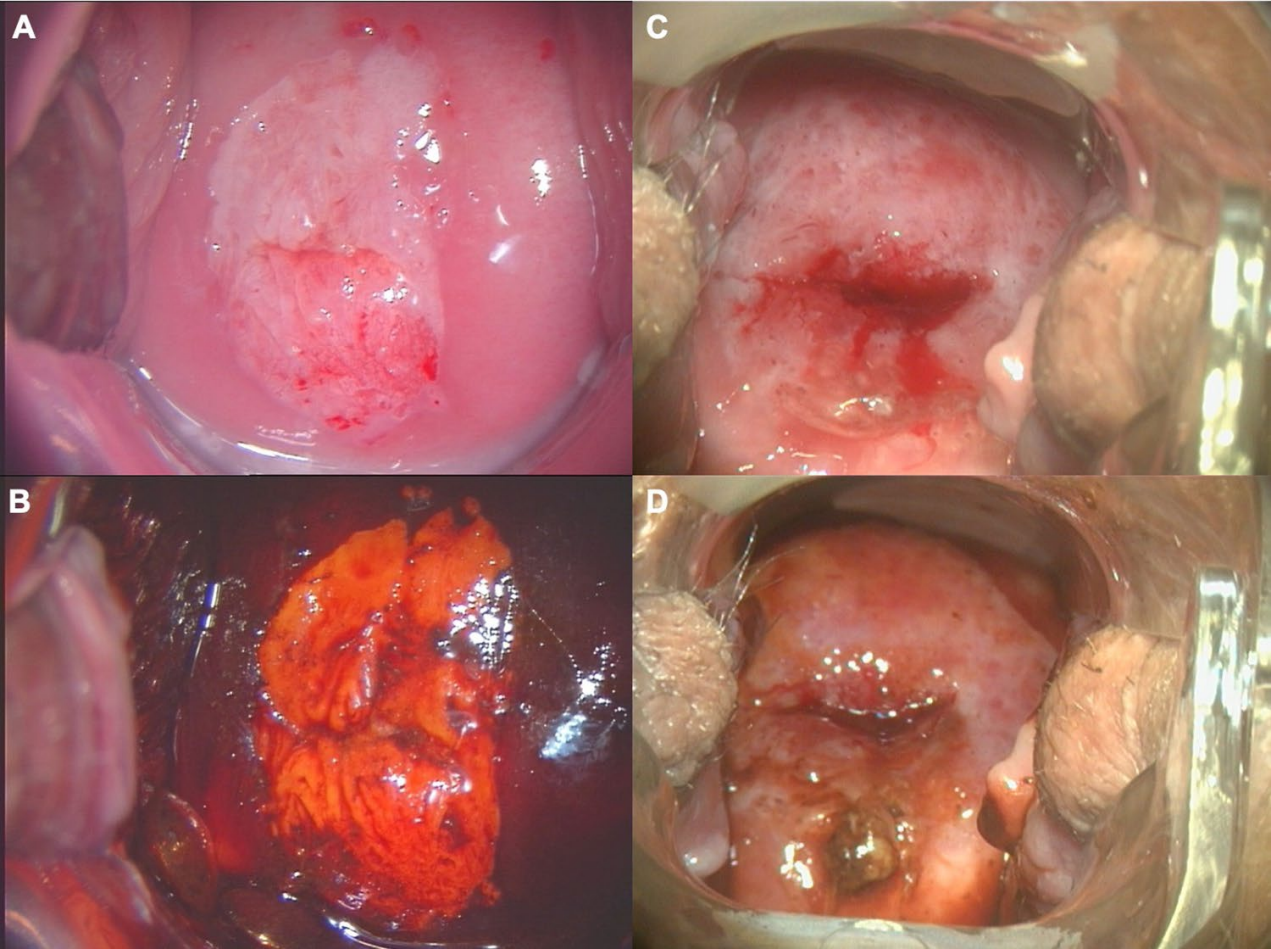
Colposcopic imaging of CIN 3. **A** + **B** CIN 3 within the borders of the cervix; LEEP possible in PCB. **C** + **D** CIN 3 reaching outside the borders of the cervix; LEEP only possible with difficulties in PCB due to extension beyond the cervix (consider general anesthesia). **A** Acid white major change in the borders of the cervix. **B** Same patient as (**A**), after after Lugol’s reaction **C** acid white major change outside the borders of the cervix. **D** Same patient as (**C**), after Lugol’s reaction

Since only the cervix is painless during PCB surgery, tight vaginal conditions are a relative contraindication. Thus, in LEEP, complete adjustability of the dysplastic area is a basic requirement. If it is not possible to adjust the area with additional specula without much inconvenience for the patient, it is advisable to perform the operation under anesthesia. Figure 4 shows examples of cervical intraepithelial neoplasia grade 3 (CIN 3) lesions which typically can be removed by LEEP in PCB and CIN 3 lesions which should rather be performed under general anesthesia due to their extension.

The most painful location during hysteroscopic entry into the uterus is the internal cervical os. Therefore, cervical stenosis is one of the most frequent reasons for aborting the operation [8].

Preoperative priming of the cervix eases entry into the cavum and allows further dilatation, which can shorten the operation time. However, significantly more side effects such as bleeding, abdominal pain, and gastrointestinal symptoms occur under this [24]. Oral and vaginal administration of misoprostol appear equivalent, but side effects such as diarrhea are less common with vaginal administration [25]. However, data on patient satisfaction and complications are lacking.

Large and well-perfused intrauterine and/or intramural localized pathologies are also relative contraindications. However, increasingly smaller instruments allow surgery of more difficult pathologies. For example, in 2018 Campo showed that the majority of hysteroscopic surgeries can be successfully performed in the office setting with ideal patient selection and optimal instrumentation [3]. With the addition of i.v. sedation, even larger pathologies can be resected in experienced hands [5].

As a general rule, surgeries in which difficulties are expected even under anesthesia should not be performed in PCB.

By using the PCB when indicated, surgeries can be moved out of the operating room and also avoid the risks of general anesthesia (e.g., PONV, POCD).

The skills needed to perform the PCB are easy to learn and will remain with us gynecologists even after the corona pandemic. The resources this frees up in the operating room can then be replaced by other surgeries. This increases the quality of care and the spectrum of gynecological minimally invasive operations can be expanded.

The additional time required to perform the PCB can be compensated for by the lack of induction, maintenance and emergence of the anesthesia; thus, the number of operations that can be performed per day remains the same while surgical resources are freed up. In addition, the costs of anesthesia (personnel, medication) can be saved and can therefore be used more efficiently elsewhere.

The postoperative monitoring time of 30 min is also significantly shorter than with general anesthesia, after which patients usually have to be monitored for between 2 and 4 h, depending on the analgesic and anesthetic medication used. Thus, scarce resources can be saved here as well.

Since the corona pandemic will continue to affect our operative gynecology for quite some time, a rethinking is also necessary in other areas of our specialty. In the near future, we will not be in a position to avoid expanding operations under local anesthesia. Other possible areas of application could be breast surgery and vulvar surgery.

## Data Availability

Not applicable.

## Abbreviations

CIN 3: Cervical intraepithelial neoplasia grade 3
LAST: Local anesthetic systemic toxicity
LEEP: Loop electrosurgical excision procedure
N_2_O: Nitrous oxide
NSAIDs: Nonsteroidal anti-inflammatory drugs
PCB: Paracervical block
POCD: Postoperative cognitive dysfunction
PONV: Postoperative nausea and vomiting
